# Quality standards for NK cell immunotherapies

**DOI:** 10.3389/fbioe.2025.1716975

**Published:** 2025-11-11

**Authors:** Valentin von Werz, Aleksander Szarzynski, Sara Zigon-Branc, Oliver Spadiut

**Affiliations:** Research Area Biochemical Engineering, Institute of Chemical, Environmental and Bioscience Engineering, Technische Universität Wien, Vienna, Austria

**Keywords:** NK cell, quality standand, ICH, CQA, clinical trial, cancer

## Quality standard landscape in NK cell therapies

Natural killer (NK) cells are innate lymphocytes capable of directly targeting and killing tumor cells without prior sensitization (Vivier et al., 2024). Due to their inherent cytotoxicity, NK cells have emerged as a very promising modality for cancer immunotherapy, offering outstanding advantages over chimeric antigen receptor-T (CAR-T) cell therapies, such as reduced risks of the cytokine release syndrome and neurotoxicity (Vivier et al., 2024; Zhang et al., 2019; Lodoen and Lanier, 2006). CAR-engineered NK cells further enhance therapeutic potential by improving target specificity and cytotoxic efficacy, thereby complementing and extending the clinical success of current CAR-T cell therapies (Vivier et al., 2024; Zhong and Liu, 2024). However, the lack of formally defined critical quality attributes (CQAs) currently limits cross-trial and cross-study comparability, compromises safety evaluation, and impedes clinical efficacy optimization for NK cell-based therapies.

CQAs play a pivotal role in biopharmaceutical development as they describe the required product quality, safety and efficacy (FDA U.S. Department of Health and Human Services, 2012). According to the International Council for Harmonisation of Technical Requirements for Pharmaceuticals for Human Use (ICH) Guideline Q8 (R2), a CQA is a “physical, chemical, biological, or microbiological property or characteristic that should be within an appropriate limit, range, or distribution to ensure the desired product quality” (ICH, 2009). Despite their central importance, CQAs have not yet been formally defined for NK cell therapies yet. The current FDA guidance only roughly defines parameters for quality testing, recommending that CQAs should minimally consist of identity, quality, purity, and potency (FDA U.S. Department of Health and Human Services, 2024; FDA U.S. Department of Health and Human Services, 2023). Regulatory agencies currently refrain from defining generalized acceptance criteria or fixed limits, as these must be adapted to each manufacturing platform, therapeutic indication, and target patient group. While clearly defined CQAs with specified ranges would bring obvious benefits, overly rigid or premature standardization could stifle innovation, particularly for next-generation therapies tailored to specific indications or specific patient populations. In contrast, the current high variance in trial release criteria is complicating product comparison and poses challenges for a consistent regulatory evaluation. We are convinced that a harmonized effort for defining CQAs - anchored in a Quality by Design (QbD) framework and aligned with ICH and regulatory guidelines - is required and essential for ensuring the consistency, safety, and efficacy of NK cell therapies.

To potentially initiate this harmonization effort, we systematically analyzed the reported release criteria from all completed Phase 1 and 2 clinical trials of NK cell therapies targeting cancer, which were conducted in the US and Europe with reported release criteria (Shah et al., 2015; McKenna et al., 2012; Lapteva et al., 2014; Dolstra et al., 2017; Williams et al., 2017; Boyiadzis et al., 2017) (Table 1).

**TABLE 1:** Suggestions for CQAs for NK cell products, based on release criteria from phase 1 and 2 cancer immunotherapy trials using primary human NK and NK-92 cells in the EU and United States. Acceptance ranges were derived from reported thresholds, with the most stringent values highlighted in bold. 4-1BBL, commonly used as a stimulatory factor on feeder cells, is listed as a contaminant, as its unintended presence may indicate feeder cell carry-over.

Category	CQA	Specification/Range
Viability	Viability	**≥70%** (Shah et al., 2015; McKenna et al., 2012; Lapteva et al., 2014; Dolstra et al., 2017; Williams et al., 2017; Boyiadzis et al., 2017)
Identity	CD56^+^	≥30% (McKenna et al., 2012) >50% (Lapteva et al., 2014) >70% (Lapteva et al., 2014; Dolstra et al., 2017) **>90%** (Shah et al., 2015; Boyiadzis et al., 2017)
	CD3^+^	**≤0.2%** (Shah et al., 2015) <3 × 10^6^ cells/kg (ICH, 2009; McKenna et al., 2012) <5 × 10^5^ cell/kg (FDA U.S. Department of Health and Human Services, 2012; Lapteva et al., 2014) <1 × 10^4^ cell/kg (Zhong and Liu, 2024; Dolstra et al., 2017)
	CD14^+^	**≤5%** (Shah et al., 2015)
	CD20^+^	**<3%** (McKenna et al., 2012)
	CD19^+^	**<3 × 10** ^**5**^ **cell/kg** (Dolstra et al., 2017)
	CD16^+^ CD3^+^	**<5%** (Boyiadzis et al., 2017)
	NKG2A^+^, NKp30^+^, NKp44^+^, NKp46^+^ and NKG2D^+^ on CD56^+^CD3^−^ cells	**>30%** (Dolstra et al., 2017)
Potency	Cytotoxicity	>20% lysis at E:T 20:1 (Lapteva et al., 2014) **32% lysis at E:T 5:1** (Williams et al., 2017) 39% at 10:1 (Zhang et al., 2019) 49% at 20:1 (Zhang et al., 2019)
Contamination	Bacteria	**Negative** (Shah et al., 2015; McKenna et al., 2012; Lapteva et al., 2014; Dolstra et al., 2017; Williams et al., 2017; Boyiadzis et al., 2017)
	Mycoplasma	**Negative** (Shah et al., 2015; McKenna et al., 2012; Lapteva et al., 2014; Dolstra et al., 2017; Williams et al., 2017; Boyiadzis et al., 2017)
	Endotoxin	**Negative** (Shah et al., 2015; McKenna et al., 2012; Lapteva et al., 2014; Dolstra et al., 2017; Williams et al., 2017; Boyiadzis et al., 2017) <0.5 EU/kg (Williams et al., 2017) <5 EU/kg (McKenna et al., 2012; Lapteva et al., 2014; Boyiadzis et al., 2017)
	Fungus	**Negative** (Dolstra et al., 2017)
	41-BBL^+^	≤1% (Shah et al., 2015) **<0,1%** (Lapteva et al., 2014)

In all the clinical trials the accepted viability range for NK cells was uniformly set at ≥70%, highlighting its importance as a benchmark linked to clinical performance. Purity was defined by the presence of other cell types in the final NK cell product. Therefore, cell types were identified by cell surface markers of NK cells (CD56^+^), T cells (CD3^+^), B cells (CD19^+^, CD20^+^), monocytes (CD14^+^), and NK-like T cells (CD16^+^CD3^+^). Activation markers (NKG2D, NKp30, NKp44, NKp46) and inhibitory receptors (NKG2A) were reported to a variable extent, and NK cell purity criteria of the final product ranged from ≥30% to ≥90% (Shah et al., 2015; McKenna et al., 2012; Lapteva et al., 2014; Dolstra et al., 2017; Boyiadzis et al., 2017). T and B cell contaminants were strictly limited between ≤0.2% and <3% (Shah et al., 2015; McKenna et al., 2012; Lapteva et al., 2014; Dolstra et al., 2017). While most studies reported acceptable purity ranges in relative units (i.e., percentage of impurities within the final product), others presented only absolute cell counts. To improve cross-study comparability and regulatory alignment, CQAs should consistently be reported in relative units. Contamination standards were uniform, requiring sterility and strict endotoxin limits (negative detection to <5 EU/kg) (Shah et al., 2015; McKenna et al., 2012; Lapteva et al., 2014; Dolstra et al., 2017; Williams et al., 2017; Boyiadzis et al., 2017) as well as the absence of feeder cell residuals, such as the stimulant 41-BBL (Shah et al., 2015; Lapteva et al., 2014). Potency assay norms also varied, with tumor cell lysis values reaching up to 49% at an effector-to-target ratio acronym: (E:T) of 20:1 (Zhang et al., 2019). However, cross-study and cross-trial comparability remain challenging, as no harmonized assay protocols, such as consistent E:T ratios or standardized target cell types, have been employed, underscoring the urgent need for assay harmonization and clear definition of CQAs.

We suggest the data summarized in Table 1 to be a potential foundation stone for the definition of CQAs with respective acceptance ranges. Defining CQAs early in development and validating appropriate assays will enhance product reliability and patient safety. Applying a QbD approach allows systematic identification and control of critical process parameters during manufacturing and their effects on CQAs. Integration of QbD principles with ICH Q8–Q11 and FDA/EMA frameworks can facilitate robust, reproducible production protocols and support smoother regulatory approvals (ICH, 2009). In our opinion, collaboration among key stakeholders, including academic researchers, biotechnology developers, manufacturers, sponsors and regulatory agencies, is essential to develop consensus guidelines and reference quality standards for NK cell products. Regulatory bodies should issue more detailed guidelines specific for NK cell products, including standardized thresholds for viability, potency, purity, and contaminant limits. While NK cells from different sources or isolation methods may display varying phenotypes, harmonized release criteria should ensure that every patient receives an NK cell product meeting certain safety, purity, and potency standards, irrespective of its origin. The proposed CQAs, derived from available clinical trial release criteria (Table 1), provide a foundational baseline for more refined, QbD-driven definition of CQA ranges tailored to specific disease indications. The analyzed clinical trial cohort was limited in size, which reflects the current scarcity of publicly available data on release criteria rather than a selective bias, underscoring the urgent need for greater transparency and standardized reporting in future NK cell trials. Harmonizing CQAs through this QbD-driven approach will be pivotal for NK cell therapy development - ensuring patient safety, optimizing clinical outcomes, and enabling more scalable, reproducible, and effective NK-based immunotherapies across diverse clinical settings worldwide.
